# Supplementary material for: Maternal body mass index and necrotizing enterocolitis: A case-control study
Source: PLoS One. 2024 Jan 24;19(1):e0296644. doi: 10.1371/journal.pone.0296644 (PMC10807840; doi:10.1371/journal.pone.0296644)
Supplement: Attachment — Submitted filename: Rebuttal leterKS.pdf [file pone.0296644.s002.pdf]

Katherine A. Stumpf, MD
Assistant Professor of Pediatrics

November 9, 2023

PLOS ONE
Editorial board

Dear Editor,

We would like to thank the reviewers for their overall positive review of our manuscript and have incorporated their thoughtful comments into this new revised manuscript. All the reviewers' points have been addressed in our updated manuscript which we summarize below and are highlighted in our manuscript:

1. Editor and reviewers: "Consider including a sentence or two on the pathophysiology behind the association of maternal BMI an NEC". **We have included an additional reference regarding the pathophysiological basis for the association of maternal BMI and necrotizing enterocolitis. This is now addressed in both the introduction and discussion sections.**
2. Editor and Reviewer 1: Please add "lack of gastrointestinal diagnoses to the methods". **We have included the reviewers' suggestion of phrasing to state "lack of a gastrointestinal diagnosis" to describe our control group in the methods section.**
3. Editor and reviewers: "Is there a way you can improve on the figures?" **We apologize that our figures were not transferred in high resolution as a pdf. Our figures have been updated in the attachments which are clearer and appropriate resolution.**
4. Editor: Change subheading. **We have changed the subheadings as requested to include both "Discussion" and "Conclusion". We have included a comment on the significance in our conclusion.**
5. Editor and Reviewer: Please provide an explanation on why you consider the number of Hispanic women to be a limitation. **An additional explanation regarding limitations of generalizability due to the large number of Hispanic women is now added to the discussion.**
6. Editor and Reviewer 1: Kindly review reference 9. **Reference 9 has been updated and completed.**

Additionally, our data set is fully available upon request and had been uploaded to the UTSW Data Repository. We believe our manuscript with the revisions suggested is significantly

5323 Harry Hines Boulevard/ Dallas, Texas 75390-9063/ Tel. 214-648-3903 Fax 214-648-2481
Katherine.stumpf@utsouthwestern.edu

improved and is now suitable for publication in PLOS ONE. Please feel free to contact us with any additional questions or concerns.

Sincerely yours,

Katherine Stumpf, MD

Assistant Professor
Department of Pediatrics
Division of Neonatal Perinatal Medicine
University of Texas Southwestern Medical Center

5323 Harry Hines Boulevard/ Dallas, Texas 75390-9063/ Tel. 214-648-3903 Fax 214-648-2481
Katherine.stumpf@utsouthwestern.edu
